# Comparison of resistance training using barbell half squats and trap bar deadlifts on maximal strength, power performance, and lean mass in recreationally active females: an eight-week randomised trial

**DOI:** 10.1186/s13102-024-00911-8

**Published:** 2024-05-31

**Authors:** Karianne Hagerupsen, Sigurd Pedersen, Nicoline B. Giller, Nora K. Thomassen, Kim Arne Heitmann, Edvard H. Sagelv, John O. Osborne, Kristoffer R. Johansen

**Affiliations:** School of Sport Sciences, Faculty of Health Sciences, UiT the Arctic University of Norway, Postboks 6050 Langnes, Tromsø, 9037 Norway

**Keywords:** Resistance training, Hypertrophy, Women, Countermovement, Sprint

## Abstract

**Background:**

The aim of this study was to investigate the effect of high load resistance training using barbell half squats compared with trap bar deadlifts on maximal strength, power performance, and lean mass in recreationally active females.

**Methods:**

Twenty-two recreationally active female participants (age: 26.9 ± 7.7 yrs.; height: 166.0 ± 5.1 cm; weight: 68.6 ± 9.9 kg) were randomly assigned to either a barbell half squat group (SG: *n* = 10) or trap bar deadlift group (DG: *n *= 12). Training consisted of twice-weekly sessions for eight weeks. Both groups completed one-repetition maximum (1RM) testing for both barbell half squat and trap bar deadlift groups. Countermovement jump (CMJ) and sprint performance were also assessed. Total body (TBLM) and leg lean mass (LLM) were measured with dual-energy x-ray absorptiometry. Between-group differences were analysed using analysis of covariance.

**Results:**

SG tended to improve 1RM half squat (21.0 ± 11.5 kg vs. 13.1 ± 7.5 kg) more than DG (mean difference (MD): 8.0 kg, 95% CI: -0.36 – 16.3 kg). A similar pattern in favour of DG (18.4 ± 11.2 vs. 11.7 ± 8.1 kg) compared to SG was observed (MD: 6.5 kg, 95% CI: -2.5 – 15.6 kg). No between-group differences for sprint, jump or lean body mass changes was observed. For groups combined, the following changes in CMJ (2.0 ± 2.4 cm), 5-m sprint (-0.020 ± 0.039 s), 15-m sprint (-0.055 ± 0.230 s), TBLM (0.84 ± 1.12 kg), and LLM (0.27 ± 0.59 kg) was observed.

**Conclusions:**

An exercise intervention consisting of half squats or trap bar deadlift were associated with improved muscle strength, power, and lean mass. Our findings suggests that in recreationally active females, exercise selection is less of a concern provided that heavy loads are applied, and relevant muscle groups are targeted.

**Supplementary Information:**

The online version contains supplementary material available at 10.1186/s13102-024-00911-8.

## Practical implications

- This is the first study to investigate if resistance training using barbell half squats, compared to trap bar deadlifts, results in greater strength and power in recreationally active females.
- Given that heavy loads and high intensity effort are applied, and relevant movement patterns are targeted, exercise selection appears to be less of a concern in order to improve sprint and jump performance in recreationally active females.
- Since barbell half squats and trap bar deadlifts are not mutually exclusive, both exercises can be incorporated into a resistance training regimen to improve maximal strength and power performance.

## Introduction

Resistance training (RT) is an important training modality that is well-established to effectively induce beneficial neuromuscular adaptations and enhance muscular health, such as increased muscle mass, strength, and physical function [1]. Furthermore, RT also improves sporting performance, for example, increased muscular strength is associated with increased force–time characteristics, such as peak force and rate of force development, and also jumping, sprinting, and sport-specific performance [2].

Previous research has established that RT with high external loads (e.g., ≥ 85% 1RM) is an effective training modality for improving power performance [3–5]. In addition to improve maximal strength and lean body mass, performance in compound movements such as the back squat and deadlift are highly correlated with jumping ability and sprint performance [3, 6–8]. Additionally, previous research with male participants suggests that deadlifting with a trap bar can result in greater force, power, and rate of force development compared to the straight barbell deadlift [9, 10]. However, despite the widespread popularity of squats and deadlifts in strength training, there is relatively little research that has investigated the comparative effect between these two exercises on strength and power performance, and furthermore females are underrepresented in relation to this topic. A recent study by Nigro et al., [11] reported that both straight barbell deadlifts and squats had similar effects on strength- and jump performance; however, this study only included male participants.

Due to the considerable differences in anthropometrical, physiological, and hormonal properties between sexes, findings from males may not be generalizable to females [12]. For example, even though females usually display similar hypertrophic responses as males, increases in relative strength may differ by sex, highlighting the need for additional female-focused RT research [12]. Only a handful of studies have examined the effect of RT on vertical jump height and sprint performance in females, with contradictory results. Some studies have reported improvements in sprint- and vertical jump performance after a period of RT [13, 14], while conversely, other studies showed no change in these performance variables [15, 16]. Thus, the effect of RT on power performance in females remain inconclusive.

The primary aim of this study was to compare the effects of twice-weekly high load RT, using barbell half squats or trap bar deadlifts, over eight weeks on strength- and power performance in recreationally active females. The secondary aim was to compare the effects of RT on lower body lean mass (LLM) and total body lean mass (TBLM).

## Methods

### Participants

A convenience sample of 24 females who were recreationally trained in RT (i.e., RT 2–3 days per week for the last 6 months) agreed to participate in the study. Inclusion criteria for the study were: females; > 18 years who were healthy and without injuries or illnesses that could interfere with strength testing- and training. Participants were recruited through social media and local announcement at university campus. Participants were randomly allocated to either a squat group (SG: *n* = 10) or a deadlift group (DG: *n* = 12) and were required to complete ≥ 70% of the training sessions to be included in the analyses. One participant did not complete the required amount of training and one participant withdrew due to an injury not related to the study. Thus, a total of 22 participants (age: 26.9 ± 7.7 yrs.; height: 166.0 ± 5.1 cm; weight: 68.6 ± 9.9 kg) completed both pre- and post-tests and were included in the final analyses.

This study was approved by the Norwegian Centre for Research Data for the storage of personal data. All participants signed informed consent forms prior to participation in the study.

### Test battery

**Fig. 1:**
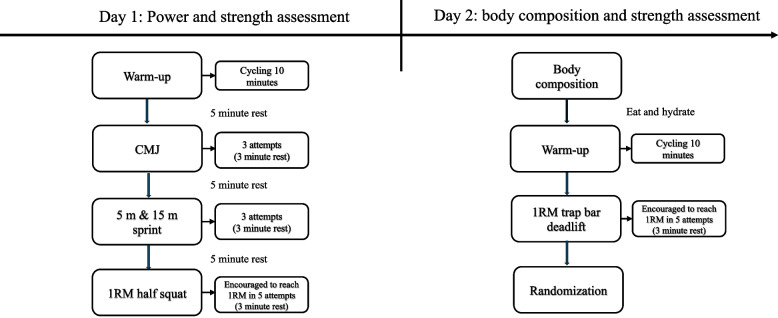
Flow chart illustrating a timeline of the testing procedures

All tests were conducted by 3rd year undergraduate students in sports and exercise science, under supervision of a trained exercise physiologist. Prior to the intervention, the participants completed a baseline test battery over two non-consecutive days. No familiarisation sessions were given for any of the test protocols prior to baseline testing. Participants were asked to refrain from vigorous exercise for the 24 h before testing. Measurements on day one consisted of countermovement jump (CMJ), 5- and 15-m sprints, and 1RM in a barbell half squat. On day two, body composition measures were recorded, followed by a 1RM test in the trap bar deadlift exercise. A timeline of the test procedures is illustrated in Fig. 1. On both test days, the participants performed the same general warm-up routine consisting of 10 min low-intensity cycling on an ergometer bike (Pro/Trainer, Watt bike Ltd., Nottingham, UK). Participants completed the same test battery again after the 8-week training intervention.

### Maximal strength

The same test procedure was used to measure 1RM in both the barbell half squat and trap bar deadlift. Barbell half squat 1RM with a 90° knee angle was carried out using a squat rack and competition standard Olympic style barbell (20 kg, Eleiko, Halmstad, Sweden). A hand-held goniometer was used to ensure ~ 90° knee angle between femur and tibia. Knee angle was assessed during a pre-warmup repetition where the participants had the barbell on their back, and then again during the first warmup set. Assessment of the 90° knee angle is illustrated in Supplementary figure S1. Maximal strength in the deadlift was measured using a trap bar (32 kg, Pivot, Sportsmaster, Norway). Previous studies have showed similar knee flexion angles in the starting position of the trap bar deadlift exercise compared to a barbell half squat [10, 17]. An image of the bottom position in the trap bar deadlift is illustrated in Supplementary figure S2. Prior to starting the 1RM attempts, participants performed four warmup sets consisting of 8–10 repetitions on 50%, 6 repetitions on 70%, 3 repetitions on 80% and 1 repetition on 90% of estimated 1RM [18]. Participants were permitted as many attempts as necessary to achieve a successful 1RM lift but were encouraged to reach their 1RM within five attempts. Load was increased by 2.5–10 kg for each successful attempt. Each attempt consisted of one repetition and separated by at least three minutes of passive rest. The heaviest weight that was successfully lifted for one repetition was reported as the participants’ 1RM.

### Countermovement jump

A force platform (MuscleLab, Ergotest Technology AS, Langesund, Norway) was used to record the CMJs. Jump height was calculated by the impulse using software that was specifically developed for the platform (MuscleLab software, v.21, Ergotest Technology AS, Langesund, Norway). Prior to the CMJ-test, the participants were given two practice jumps. All participants then performed three jumps with their hands placed on the hips, with a self-selected depth for the countermovement. Each attempt was separated by minimum three minutes of rest. The highest jump was recorded as their CMJ height and carried forward for final analyses.

### 5-m and 15-m sprint time

Sprints were completed on an indoor field with artificial grass. Single-beam photocells (ATU-X, IC control AB, Stockholm, Sweden) mounted to the wall at the start, 5-m, and 15-m distances were used to record the sprint times. Prior to the sprint tests, the participants performed two 15-m practice sprints at approximately 80% of self-perceived maximum speed. Participants started 30 cm behind the first photocell and triggered the timer to start recording when breaking the sensor beam. Participants self-selected when to start the sprint and completed three attempts, each separated by a minimum of three minutes of rest. The fastest 5- and 15-m sprint times were used for the final analyses.

### Body composition

TBLM, LLM and body fat percentage were measured pre- and post-training intervention with dual-energy x-ray absorptiometry (DXA) using the Lunar Prodigy Advance (GE Medical Systems, Madison, Wisconsin, USA), operating the enCORE software (GE Medical Systems, Madison, Wisconsin, USA). All participants received a whole-body scan according to the manufacturer´s guidelines. Participants were wearing underwear, without shoes, and were asked to remove all jewellery and other personal effects that could interfere with the measurement. As hydration status influences body composition measurements using DXA [19], participants were instructed to refrain from food and drinks for 12 h before the test. Participants were allowed to eat and hydrate before proceeding with the strength test. LLM was established by manual placement of subregions of interest based on anatomical landmarks. It was defined as the area from the femoral neck to the malleolus lateralis, as described by Midorikawa et al. [20].

### Training protocol

**Fig. 2:**
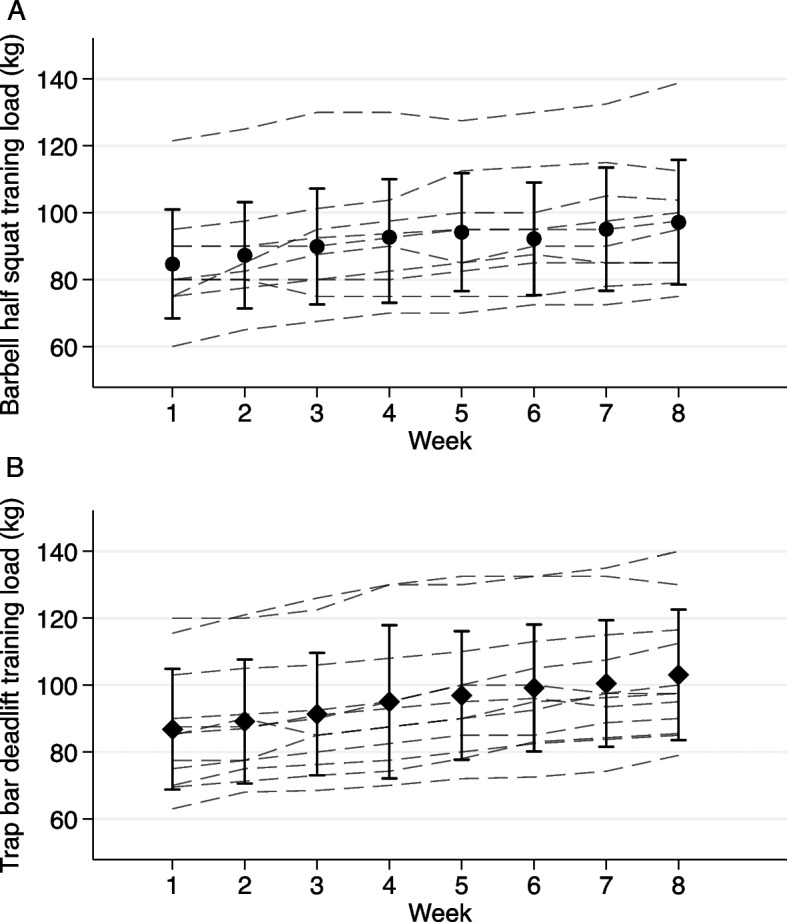
Training log for the barbell half squat exercise in SG (**A**) and trap bar deadlift exercise in DG (**B**). The circles (**A**) and diamonds (**B**) represent the average weight lifted ± SD each week. The dashed lines represent individual observations

After baseline-testing, participants attended non-supervised training twice a week for 8 weeks. The DG were instructed to perform four sets of four repetitions of trap bar deadlifts, while the SG performed the same number of sets and repetitions with barbell half squats. The load was initially set at 85% of pre-test 1RM. Participants were instructed to increase the load by 2.5 kg to 10 kg if they could complete more than four repetitions, resulting in consistent, progressive overload during the training intervention. If the participants were unable to execute all four repetitions successfully, the weight was lowered by 2.5 kg to 5 kg on the next set. Participants were also instructed to perform each repetition with maximal intended velocity in the concentric phase. The average weight lifted in each session was logged by all participants during the intervention (Fig. 2). Both groups were additionally tasked with performing 3 sets of 8 repetitions of both Bulgarian split squats using dumbbells and barbell hip-thrusts, during every training session. For these additional exercises, two repetitions in reserve were used as the target intensity. An overview of the training protocol is presented in Table 1. Participants were instructed to start every training session with their specific group target exercise (i.e., squats or trap bar deadlift), and then perform Bulgarian split squats and hip thrusts in whatever order they preferred. Each set was separated by ≥ 3 min of rest for all exercises. Participants were instructed to refrain from any other lower-body strength and/or power training during the 8-week intervention period, whereas other exercises were permitted (e.g., upper-body strength training and endurance training).

**Table 1:** Resistance training protocol

	**Exercise**	**Sets**	**Repetitions**	**Intensity**
**SG**	Squat	4	4	> 85% 1RM
**DG**	Trap-bar deadlift	4	4	> 85% 1RM
**Both groups**	Bulgarian split squat	3	8	2 RIR
	Barbell hip-thrust	3	8	2 RIR

*SG* Squat group, *DG* Deadlift group, *1RM* One repetition maximum, *RIR* Repetitions in reserve

### Statistical analyses

All analyses were performed using Stata (v17; StataCorp LLC, Texas, United States). Normality for all variables was confirmed using Shapiro Wilk tests and visual inspection of QQ-plots. The post-test difference between-groups was assessed with an analysis of covariance (ANCOVA). The post-test value was modelled as the outcome, with the variable “group” entered into the model as a factor, and the pre-test result as a continuous covariate (Y_posttest_ = β_1Group_ + β_2pretest_). This procedure may yield higher statistical power and potentially more valid results compared to a simple comparison of post-test differences or an evaluation of differences in change scores [21]. In addition, for graphical purposes, we used the same model but with change scores as the outcome (Y_Δ_ = β_1Group_ + β_2pretest_) which has been reported as equivalent to an ANCOVA when change scores are adjusted for baseline values [22]_._ Model assumptions were assessed by inspecting residual versus predictor plots and performing White´s test of heteroscedasticity. In addition, when no apparent differences (or tendencies) between groups were observed, we reported pre- to post-test differences for both groups combined, for simplicity. The level of statistical significance was set at α = 5%. Descriptive data are presented as mean ± standard deviation, and modelled outcomes as adjusted means with corresponding 95% confidence intervals (CI). Furthermore, pre- to posttest changes within groups, or for groups combined are reported with descriptive statistics only and without a corresponding null hypothesis test because comparisons against baseline can be highly misleading [23].

## Results

In total, 22 participants completed both pre- and post-tests, and the mean training adherence across the 8-weeks for SG and DG were 88% and 93%, respectively. One participant in the DG was excluded from the CMJ analyses due to equipment error on the post-test. Five participants, three in the SG and two in the DG, were excluded from the LLM analyses due to equipment error.

**Fig. 3:**
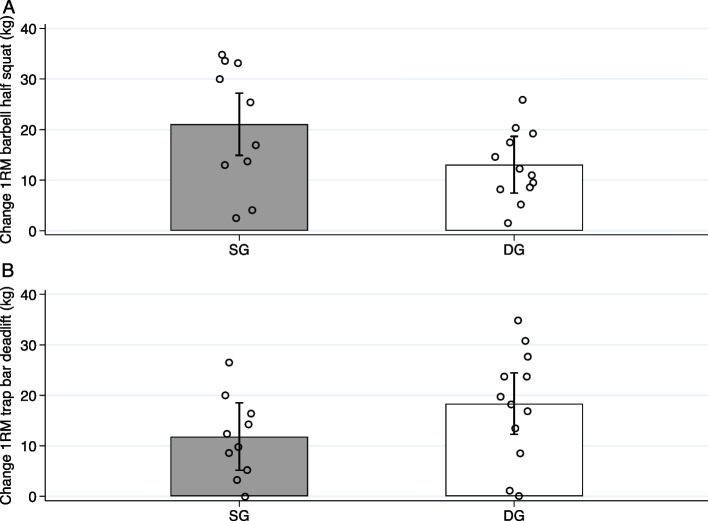
Change in 1RM barbell half squat (**A**) and trap bar deadlift (**B**) from pre- to post-test. Data are presented as mean change ± SD. Scatter dots represent individual observations. SG, Squat group, DG, Deadlift group, 1RM, one repetition maximum

**Fig. 4:**
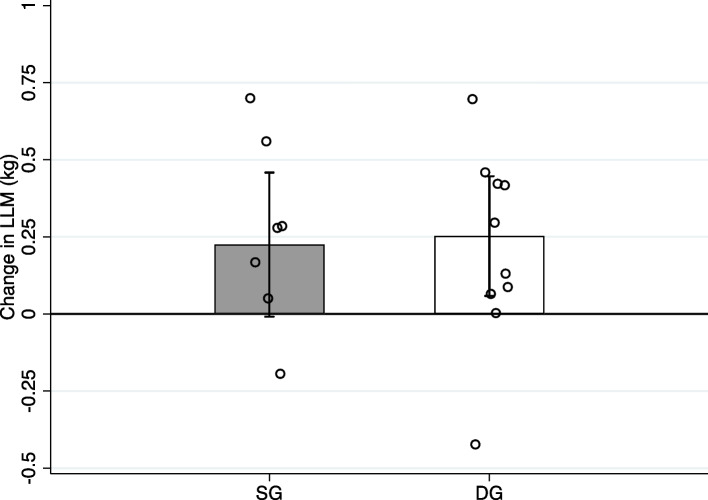
Change in LLM from pre- to post-test. Data are presented as mean change ± SD. Scatter dots represent individual observations. SG, Squat group, DG, Deadlift group, LLM, leg lean mass

There were no significant differences between the groups for 1RM in either barbell half squat (*p* = 0.059) or trap bar deadlift (*p* = 0.146; Table 2). Furthermore, we observed no between group differences for change in CMJ performance (*p* = 0.919), 5-m sprint (*p* = 0.562), 15-m sprint (*p* = 0.568), TBLM (*p* = 0.773) or LLM (*p* = 0.848). Changes in 1RM and LLM are presented in Figs. 3 and 4.

**Table 2:** Differences in strength- and power performance

	**SG** (*n* = 10)		**DG** (*n* = 12)		
**Performance metric**	**Pre**	**Post**	**Pre**	**Post**	**Adjusted mean difference (95% CI)**
Total body mass (kg)	69.3 ± 10	69.3 ± 9	68.0 ± 10	69.1 ± 11	0.92 (-2.20 – 4.03)
Body fat (%)	30.1 ± 6	29.8 ± 5	31.6 ± 7	30.8 ± 7	-0.51 (-1.69 – 0.68)
TBLM (kg)	44.9 ± 4	45.8 ± 4	43.7 ± 4	44.5 ± 4	-0.15 (-1.20 – 0.91)
LLM (kg)^a^	17.2 ± 2.1	17.4 ± 2.3	16.3 ± 1.1	16.5 ± 1.4	0.03 (-0.28 – 0.34)
Sprint time (s)					
5-m	1.12 ± 0.08	1.10 ± 0.07	1.11 ± 0.07	1.10 ± 0.08	0.01 (-0.03 – 0.05)
15-m	2.86 ± 0.18	2.84 ± 0.14	2.80 ± 0.17	2.79 ± 0.18	-0.01 (-0.07 – 0.06)
CMJ (cm)^b^	28.9 ± 5.4	30.9 ± 5.1	28.7 ± 4.3	30.7 ± 5.7	0.11 (-2.22 – 2.44)
1RM 90° squat (kg)	101 ± 18	122 ± 19	100 ± 23	113 ± 21	-8.00 (-16.34 – 0.36)
1RM trap bar deadlift (kg)	102 ± 14	114 ± 19	104 ± 21	122 ± 23	6.54 (-2.50 – 15.58)

Pre and post values are observed data presented as mean ± SD. Differences between groups at post-test are presented as adjusted mean difference with 95% confidence interval (CI) with SG as the reference group

*SG* Squat group, *DG* Deadlift group, *TBLM* Total body lean mass, *LLM* Leg lean mass, *CMJ* Counter-movement jump, *1RM* One repetition maximum, *CI* Confidence interval

^a^3 participants in the SG and 2 participants in the DG were excluded from the LLM analysis

^b^1 participant in the DG was excluded from the CMJ analysis

1RM in barbell half squats increased by 20.8% (21.0 ± 11.5 kg), and 13.0% (13.0 ± 7.5 kg) for SG and DG, respectively. 1RM in trap bar deadlift increased by 11.7% (11.8 ± 8.1 kg) and 17.3% (18.3 ± 11.2 kg) for SG and DG, respectively.

For the whole cohort, CMJ height, 5-and 15-m sprint time, TBLM and LLM changed by 6.9% (2.0 ± 2.4 cm), 1.8% (-0.020 ± 0.039 s), 0.7% (-0.055 ± 0.231 s), 1.9% (0.84 ± 1.2 kg) and 1.6% (0.27 ± 0.59 kg), respectively (Table S1).

## Discussion

This study compared the effect of eight weeks of twice-weekly training with either barbell half squats or trap bar deadlifts on strength and power performance in recreationally trained females. No between-group differences in the magnitude of improvement for maximal strength, sprint- or jump performance was found. Moreover, no differences in measures of lean mass (i.e., TBLM and LLM) were identified between the two groups. These findings demonstrate that there is a considerable cross-over effect when regularly training with either barbell half squat or trap bar deadlift, leading to considerable increases in 1RM for both exercises.

This study is, to our knowledge, the first to compare barbell half squats with trap bar deadlifts, using high external loads, in recreationally active females. The results indicate that both exercises are highly effective at inducing substantial strength adaptations (+ 13.0 − 20.8% 1RM) over a relatively short 8-week timeframe, which aligns with similar findings reported for trained male athletes [3, 24]. Although there were no significant between-group differences for 1RM, unsurprisingly, both groups tended to improve more in the exercise prescribed for the intervention. Strength increases specific to exercise allocation were to be expected due to training specificity [25].

The primary underlying mechanisms driving the observed strength increases were likely neuromuscular adaptations, which are particularly pronounced during the first several weeks of strength training, especially in less experienced participants [12, 25]. However, we observed a slight increase in TBLM and LLM in both groups, suggesting that morphological changes, such as increased muscle fiber size, may have also occurred. Although morphological adaptations to RT are usually evident after more prolonged training periods (i.e., > 8 weeks) [25], some studies have reported increased hypertrophy in females within shorter timeframes [13, 26, 27], which align with our results. As only one of these previous studies used DXA to measure changes in body composition [27], our results provide additional supportive evidence for the occurrence of hypertrophy in females following short RT protocols.

The strong association between back squat strength and jump- and sprint performance is widely reported for males [8, 28, 29], whereas similar data for females remains conflicting [13–16]. One potential reason for these equivocal findings may be the focus on researching team-sport athletes, who often continue to undertake similar sport-specific movements (i.e., jumps and sprints) during their regular training, potentially confounding the study outcomes. However, the participants in our study were not involved in any team-sport activities, and as such, did not perform these sport specific movements. It is therefore likely that the adaptations observed in this study were due to the RT intervention. Both groups showed similar improvements in jump- and sprint performance, indicating that high load RT with either barbell half squats or trap bar deadlifts can improve power performance in females. This study used high external training loads (≥ 85% 1RM), which presumably leads to neuromuscular changes [25]. Several factors could possibly contribute to the observed changes in jump- and sprint performance, such as improved motor unit recruitment, firing frequency, and intramuscular coordination [25], although the exact reason(s) remain unclear since in-depth neurophysiological measures were not collected. Future research should consider collecting such data to provide insights into proposed neuromuscular adaptations.

Previous research has reported that RT significantly improves sprint- and jump performance of adolescent sub-elite female football players [14] and untrained collegiate females [13]. In contrast, no significant performance improvements have been found for elite level female football players [15, 16]. This inconsistency is likely due to differences in training status and experience between the participants of these studies, as weaker and younger individuals do not usually possess optimal strength levels for expressing high power outputs [2]. Thus, increased lower-body maximal strength capacity may potentially also lead to improved power performance in these individuals [2, 4]. This suggests that participants with less training experience could increase jump- and sprint performance with high load RT, and as their training status improves, more specific training is needed for further improvements [2].

The present study provides important insight and contributes to increasing the limited existing knowledge base regarding female strength- and power performance. Another strength of our study is the use of DXA to measure body composition. Although DXA is sensitive to hydration status, it has been shown to have high accuracy and precision for estimating lean mass and fat mass and has been used for body composition assessment in a wide range of studies [19]. Furthermore, 1RM, photo cells and force platforms are considered gold standards for measuring maximal strength, sprint and jump performance [18].

This study also had several limitations. For example, there was no familiarization period before the training intervention, which could partly explain the improvements seen in this study (i.e., a learning effect). The use of unsupervised training may have also attenuated training intensity and therefore slowed the rate of progression. Thus, supervision for each training session may have potentially led to greater strength improvements [30]. The additional exercises included in the training program may also have contributed to the observed improvements in sprint performance. Previous research has shown improved sprint performance with RT using the hip thrust exercise [14, 31]. Moreover, the additional exercises also increase total training volume, which may have contributed to the improved 1RM, TBLM, LLM and power performance, and thereby potentially masking differences in the outcome variables. However, the inclusion of these accessory exercises arguably enhances the study’s external validity and practical relevance, as multiple exercises in a training session reflects a ‘normal’ training program [1]. Furthermore, only nine participants (SG = 3; TG = 6) completed 100% of the training sessions, indicating that the remaining participants had weeks with one, or no, training sessions. The participants’ menstrual cycle was unfortunately not recorded in the present study which is also a limitation. However, studies have suggested likely trivial to no influence of menstrual cycle on training adaptations [32], and thus are unlikely to have altered the response. Limitations of our sample size likely contributed to the somewhat imprecise estimates in our results. Given our somewhat limited sample, this study likely had a low statistical power to detect small and medium effect sizes which increases the type II error probability. However, given that no difference between groups in terms of lean tissue mass as well as sprint and jumping performance would be expected, an increased sample size would perhaps have been unlikely to change our conclusions.

## Conclusions

The present study suggests that twice-weekly RT sessions with high external loads (≥ 85% 1RM), using either half squats or trap bar deadlifts, can significantly increase lower-body maximal strength and enhance power performance in recreationally active females over a period of eight weeks. The results of this study also suggest that both exercises can be used to increase leg lean mass in this population. Thus, our findings suggest that if high intensity effort with high loads are emphasized, and relevant muscle groups are targeted, selection of either squats or trap bar deadlifts is less of a concern in recreationally active females aiming to improve their muscle strength, power, and lean mass.

### Supplementary Information

Supplementary Material 1.

Supplementary Material 2.

Supplementary Material 3.

## Data Availability

All relevant data used in this study are available upon reasonable request to the corresponding author.

## Abbreviations

RT: Resistance training
SG: Squat group
DG: Deadlift group
1RM: 1 Repetition maximum
CMJ: Counter movement jump
TBLM: Total body lean mass
LLM: Lower body lean mass
