# Supplementary material for: Comparison of resistance training using barbell half squats and trap bar deadlifts on maximal strength, power performance, and lean mass in recreationally active females: an eight-week randomised trial
Source: BMC Sports Sci Med Rehabil. 2024 May 31;16:124. doi: 10.1186/s13102-024-00911-8 (PMC11140948; doi:10.1186/s13102-024-00911-8)
Supplement: Supplementary file 1 — Supplementary Material 1. [file 13102_2024_911_MOESM1_ESM.docx]

**Supplementary materials**


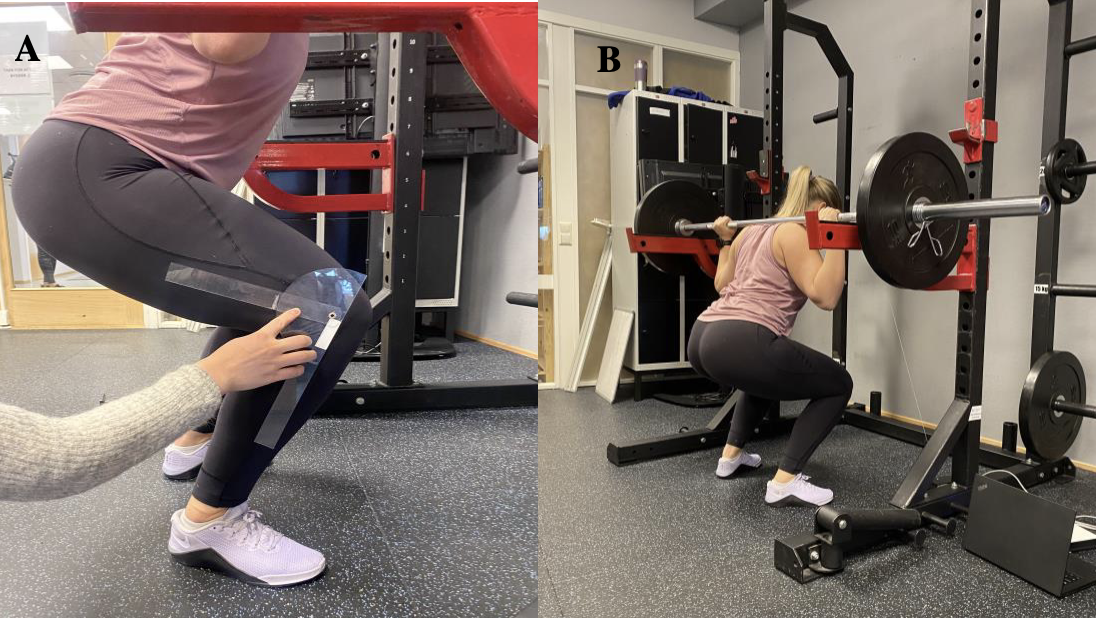


**Figure S1**. Illustration of knee angle assessment for the barbell half squat using a goniometer with a 90° knee angle between femur and tibia (A). Illustration of a participant in the bottom position of a barbell half squat (B).
