# Supplementary material for: Comparison of resistance training using barbell half squats and trap bar deadlifts on maximal strength, power performance, and lean mass in recreationally active females: an eight-week randomised trial
Source: BMC Sports Sci Med Rehabil. 2024 May 31;16:124. doi: 10.1186/s13102-024-00911-8 (PMC11140948; doi:10.1186/s13102-024-00911-8)
Supplement: Supplementary file 2 — Supplementary Material 2. [file 13102_2024_911_MOESM2_ESM.docx]

Supplementary materials


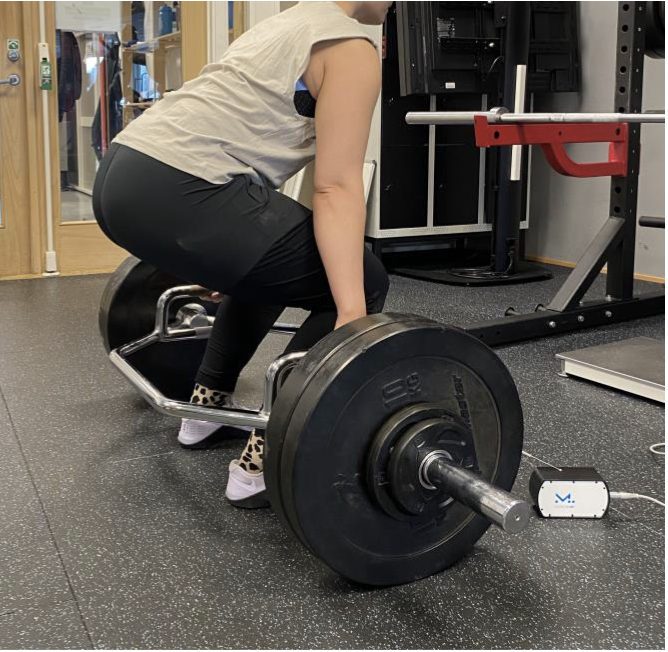


**Figure S2**. Illustration of a participant in the bottom position of the trap bar deadlift
