# Supplementary material for: Comparison of resistance training using barbell half squats and trap bar deadlifts on maximal strength, power performance, and lean mass in recreationally active females: an eight-week randomised trial
Source: BMC Sports Sci Med Rehabil. 2024 May 31;16:124. doi: 10.1186/s13102-024-00911-8 (PMC11140948; doi:10.1186/s13102-024-00911-8)
Supplement: Supplementary file 3 — Supplementary Material 3. [file 13102_2024_911_MOESM3_ESM.docx]

**Supplementary materials**

**Table S1.** Changes in strength and power performance for both groups combined.

|  | **Both groups** (n=22) | |
| --- | --- | --- |
| **Performance metric** | **Pre** | **Post** |
| Total body mass (kg) | 68.6 ±10 | 69.2 ±10 |
| Total body fat (%) | 30.9 ±7 | 30.4 ±6 |
| TBLM (kg) | 44.3 ±4 | 45.1 ±4 |
| LLM (kg)^a^ | 16.6 ±2.1 | 16.9 ±2.3 |
| Sprint time (s) |  |  |
| 5-m | 1.12 ±0.08 | 1.10 ±0.07 |
| 15-m | 2.83 ±0.183 | 2.81 ±0.14 |
| CMJ (cm)^b^ | 28.8 ±5.4 | 30.8 ±5.1 |
| 1RM 90° squat (kg) | 100 ±20 | 117 ±20 |
| 1RM trap bar deadlift (kg) | 103 ±17 | 117 ±22 |

Data are presented as mean ± SD. TBLM, total body lean mass LLM, leg lean mass, CMJ, Countermovement jump, 1RM*,* One repetition maximum, CI, confidence interval. ^a^5 subjects were excluded from the LLM-analyses. ^b^1 subject was excluded from the CMJ analysis.
